# Are fundamental niches larger than the realized? Testing a 50-year-old prediction by Hutchinson

**DOI:** 10.1371/journal.pone.0175138

**Published:** 2017-04-12

**Authors:** J. Soberón, B. Arroyo-Peña

**Affiliations:** Biodiversity Research Center and Department of Ecology and Evolutionary Biology, University of Kansas, Lawrence, Kansas, United States of America; University of Porto, PORTUGAL

## Abstract

For more than 50 years ecological niches have been defined as combinations of multidimensional environmental conditions permitting a species to survive and reproduce. A fundamental niche (**N**_F_) is defined as the set of conditions within which a species can live in the absence of competitors, and a realized niche (**N**_R_) is a **N**_F_ hypothetically reduced by competitive interactions (and some other limiting factors). This definition implies that **N**_F_ is “larger” than **N**_R_, something that has been nearly universally accepted by ecologists. However, there have been few attempts at empirical tests. Here, we present a novel quantitative test using one-dimensional estimates of **N**_F_ for 105 species of reptiles and amphibians, and estimates of **N**_R_ obtained from ~1.4 x 10^4^ field observations. To specify our test, we operationalize the original classification of niche types. Our results predominantly support the hypothesis that **N**_F_ ‘is larger’ than **N**_R_ and we highlight the theoretical and practical importance of quantifying niches.

## Introduction

The fundamental niche (**N**_F_) of a species is determined by its physiological range of tolerance to environmental factors in the absence of biotic interactions [1–3], and the regions of the planet with environments in **N**_F_ would represent some sort of potential area of distribution for the species. However, the presence of competitors and predators [4], and dispersal limitations of the species [5] reduce the occupied geographic range from its full potential, suggesting a partial occupation of available **N**_F_. Furthermore, limited climate availability [6] is also expected to reduce **N**_F_ to a smaller realized niche (**N**_R_). On the other hand, mutualistic interactions should have the opposite effect [3, 7–10].

1. Hutchinson [1] first proposed the idea that **N**_F_ should in some sense be larger than **N**_R_. Although old and superficially obvious, Hutchinson’s idea has seldom been tested, partially because the concept of “niche” originally combined many types of variables that caused not only terminological imprecision [11] but also serious complications at measuring the fundamental niche, mostly when the variables used to define it are resources consumed by the species in question [11]. Here, we use the Grinnellian niche concept [10, 12] in which niche space is defined using non-interactive conditions (i.e., scenopoetic variables [13]); specifically, we use one climatic variable (average monthly temperature) measured at every cell of a discrete geographic grid of the planet (**G**). Although we restrict our example to a niche defined by a single scenopoetic variable, all the computations can be performed using more variables, as we show in S1 File, Section 2: Estimation of niches in two dimensions.

Data for niches defined for climatic variables are substantially more available [7] than those based on interactive variables, like resources [12, 14, 15], and permit establishing straightforward relationships between metrics in environmental and geographic spaces [16–18]. For climatic variables, Hutchinson’s idea can be interpreted as a set inequality: **N**_*F*_ ⊇ **N**_*R*_, which predicts that climates in localities where a species has been observed (**N**_R_) should be located inside the geometric shape defining its **N**_F_. Hutchinson (1957) chose as shapes simple rectangles. This can be empirically true or false, since the environments where a species is observed may be outside the shape defining its fundamental niche, for a variety of reasons discussed below. We stress that the inequality is interpreted as *points* being inside a *shape*.

Although Hutchinson [1] briefly mentioned that not every environment in a fundamental niche may actually exist at a given time and space, this very important insight was essentially ignored until Jackson and Overpeck [10] rediscovered it in the context of paleoclimatic effects on vegetation. The “existing niche”, denoted by N*(*t*, **G**), is the intersection (in the sense of points being inside shapes) of the fundamental niche, which is modeled as some geometric shape, with the discrete set of environments available at time *t* in region **G**, (including the points where observations of the species has taken place) henceforth denoted by **E**(*t*, ***G***). This intersection generates the subset of the existing environmental space that is suitable for a species. **E**(*t*, ***G***), a set of points, can be modelled continuously using smooth kernels (see S1 File, Fig A1). Then, the existing niche can be added as an additional term to Hutchinson’s inequality as follows [19]:

$$N_{F}⊇N^{*}(t,G)=N_{F}\cap E(t,G)⊇N_{R}(t,G)$$ (1)

There is a correspondence between points in environmental space and the cells in the grid **G** called the Hutchinson’s Duality [7, 18]. The correspondence is not necessarily one to one [16] but it can be made to be using enough independent variables and good precision. This correspondence allows a spatial interpretation of Eq (1). First notice that although **N**_F_ *per se* cannot be mapped geographically (it is a physiological feature of a species), both N*(*t*, **G**) and **N**_R_(*t*, **G**) can, since they are sets of points in environmental space corresponding to geographic localities [2, 7, 18]. Therefore, the inequalities Eq (1) have implications for both niches and distributional areas: (i) the physiology of a species determines the potential geographical limits of its distribution (at a given time), by defining the regions with climates within its limits of tolerance. However, the actual occupied area is the result of other factors that probably constrain this potential. **N**_R_(*t*, **G**) thus corresponds to the climates in the actual species distribution [7]. (ii) The inequality Eq (1) sets the limits of niche modeling because the output of presence-only correlative distribution models can be interpreted to be approximately within the potential and occupied areas [20]; therefore, a niche modeled using correlative methods is probably intermediate between N*(*t*, **G**) and **N**_R_(*t*, **G**). (iii) Because the data used in niche modeling algorithms are samples of **N**_R_(*t*, **G**), the inequality highlights that algorithms that very faithfully model such samples may be poor at estimating **N**_F_. (iv) In niche evolution, **N**_F_ is the unit upon which evolution acts [21]; however, **N**_R_(*t*, **G**), which is estimated by correlative methods may change in position or shape due to a combination of evolutionary, ecological, and climate-availability variables mentioned above [19]. In particular, the fundamental niche is being “distorted” all the time by the shape of the existing climate in a particular region. Therefore, the inequalities Eq (1) implies that observing niche differences among regions should be possible even in the absence of niche evolution in a strict sense [22]. And finally, (vi) when applied to economically important species (invasive, forestry, vectors of diseases), the inequalities suggest that regions with environments suitable for potential introduction of such species may be substantially larger than what correlative models, based on **N**_R_(*t*, **G**) data, may predict [23]. The above factors indicate the importance of testing empirically Hutchinson’s inequalities, and it is surprising that so few attempts have been made to do it [15, 23, 24].

Notice also that the inequalities Eq (1), which describe sets, suggest inequalities on the sizes of such sets. Using vertical bars to denote a measure on the size of the sets we can hypothesize:

$$\left|N_{F}\right|\geq \left|N^{*}(t,G)\right|\geq \left|N_{R}(t,G)\right|$$ (2)

And since in principle it is possible for the realized niche to be outside the fundamental, inequalities Eqs (1) and (2) are to a degree independent of each other.

One of the reasons why few attempts have been made to test Hutchinson’s inequalities is that information on the multivariate fundamental niche is extremely rare. To test the inequalities one needs data on the physiologically defined limits of tolerance of species to extreme values of relevant niche variables. These are mostly available for one variable: temperature. In this work we will use as proxies for **N**_F_ (actually, a projection of it in one dimension), data on the lower critical temperature for reptiles and amphibians, and the upper lethal temperature for amphibians and upper critical for reptiles (see Methods), compiled by Sunday et al. [25]. In the supporting information, and for the purpose of illustration, we present calculations based on two dimensional ranges of tolerance, calculations that can be generalized to more dimensions, if data were available (S1 File, Section 2).

Using a single variable to model the fundamental niche is a serious assumption when testing Eq (1), because a multidimensional cloud of points is projected into a line, and only one inequality is tested. The observed points may be outside the inequalities in variables not included, but inside the inequality used. This is a very important caveat, but we cannot see a way out unless experimental information about **N**_F_ in higher dimensional spaces become available in the future.

To estimate **N**_R_(*t*, **G**), records of observed field occurrences were obtained from the Global Biodiversity Information Facility (GBIF) [www.gbif.org]. Because environments in these points are instances of the realized niche, Hutchinson’s inequality predicts that the environments at the occurrence points of a species should be inside its **N**_F_ range. To estimate **E**(*t*, **G**) for the world at present, and thus N*(*t*, **G**), we used WorldClim data (see Methods). To test Eq (2) we used numerical integration on smooth kernels calculated around the data points representing N*(*t*, **G**) and **N**_R_(*t*, **G**). The analysis clarifies the meaning of Hutchinson’s inequalities and provides unequivocal support for them.

Comparing macroclimatic data to critical temperatures obtained in the laboratory has a number of problems, since the temperature that individuals experience in the field may correlate poorly with macroclimate measurements [26]. One way of dealing with the problems is by using soil, wind, cloud cover, micro topography and habitat information to parameterize macro-to-micro-climate models [27]. Unfortunately, as of today, lack of data for most regions prevents the method from being generally applicable. On the other hand, many macroecological (i.e., coarse spatial resolution) patterns are assumed to be the result of interactions between macroclimate and physiology [28, 29], and for the problem of understanding how physiological limits affect geographic distributions, long-term climatic averages may matter more [30]. Indeed, many recent studies combine macroclimatic and physiological data without modeling microclimate [31–33]. Finally, since the most likely effect of microclimatic variation would be to add favorable spots to spatial units that appear climatically unfavorable at low resolutions [34], by ignoring microclimatic complications we will be erring on the conservative side, because cells unfavorable at the macroclimate level but with documented occurrences due to favorable microclimates will be misclassified.

## Methods

To obtain occurrence climates, the emerged Earth’s surface was divided into a 10^4^ km^2^ per-cell grid using the Behrmann equal area projection [35]. The resulting surface (excluding Antarctica) was represented by 16,712 cells. Present data of mean temperature were obtained for each cell by applying the ‘extract’ function in the ‘raster’ R package [36] to climates in WorldClim [37] at 10’ of resolution (~3.4 x 10^2^ km^2^ near the equator), to extract the variable Bio1 (mean monthly temperature X 10). Using the function SmoothKernelDistribution of *Mathematica*^™^, a smooth kernel *kE*(*T*) was fitted to the present E-space (Bio1/10) (Fig A1 in S1 File).

Using the ‘gbif’ function contained in the dismo R package [38], and the ‘thin’ function in the spThin package, and scripts developed in R, we queried the Global Biodiversity Information Facility (GBIF, www.gbif.org), obtaining ~76,000 coordinates for 105 names, out of the 158 originally in the Sunday et al. [25] database. We kept only 14,051 non-redundant (at 10 km) [39, 40] georeferenced records from this database (S1 File, Fig A2). Obvious georeferencing problems were detected by checking the presence of GBIF records within the recorded borders of countries (Environmental Systems Research Institute; http://www.esri.com/) and excluding them from the database. The ‘extract’ function in the ‘raster’ R package [36] was applied to coordinates in the GBIF occurrences, to obtain values of variable Bio1 in WorldClim to be divided by10.

We defined the fundamental niches of each species as the set of temperatures contained in the interval between the limits of physiological critical and/or lethal temperatures $N_{F_{j}}(x)=\{T|T_{\text{min}}\leq T\leq T_{Max}\}$ for 151 species of reptiles and amphibians, obtained from Sunday et al. [25], who give details of these definitions. The measure of **N**_F*j*_ for species *j* is simply its range $\left|N_{F_{j}}|=(T_{\text{max}}-T_{\text{min}}\right)$. To have a common measure for comparison, the extracted values of Bio1/10, per species, were standardized to a common scale of fundamental niches using ${\overset{⌢}{T}}_{i,j}=\frac{\left(Bio1_{i,j}/10-T_{Min}{}_{j}\right)}{\left(T_{Max}{}_{j}-T_{Min}{}_{j}\right)}-\frac{1}{2}$, where ${\overset{⌢}{T}}_{i,j}$ is the standardized *i*-th observation of temperature, for species *j*, and *T*_*min j*_ and *T*_*max j*_ are the lethal minimum and maximum temperatures, respectively, for species *j*.

To obtain a null model for the distribution of **N**_F_ volumes, we randomly generated 10,000 left and right limits within the range of values of Bio1 in the current climate and calculated the range value for pairs with positive Δ_*T*_ = (*Bio*1_*Max*_−*Bio*1_*Min*_)/10.

On a species per species basis, we counted the number of times that the temperature at species occurrences (GBIF) was inside their **N**_F*j*_. Significance testing required estimating the probability that random points from the E cloud will be located inside an **N**_F*j*_ range. The limits of the critical/lethal temperatures of each species (the fundamental niches) were used to integrate numerically the kernel of climate, yielding the proportion of the kernel inside every **N**_F_, which is a proportional measure of the existing niche: $|N_{F_{j}}^{*}|=\int_{T_{{\text{min}}_{j}}}^{T_{{\text{max}}_{j}}}kE(x)dx$. This is an estimate of the proportion of **E**(*t*, **G**) located inside a particular **N**_F_, or $N_{j}^{*}(x)=\{x\in E(x)|T_{{\text{min}}_{j}}\leq x\leq T_{{\text{max}}_{j}}\}$. Assuming random placement of points in the E-space, for species *j*, the probability of having *k*(*j*) points inside the *j*-th **N**_F_, out of *n*(*j*) occurrences, is binomial with probability $\left|N_{F_{j}}^{*}\right|$. Thus, for each of the 105 species, we tested the significance of the number of observed points inside **N**_F_ assuming random placing. A sequential 0.01 level of significance Bonferroni correction [41] was used.

It is known that climatic variables at coarse resolution can underestimate the amount of favorable environments in habitats protected by topographic or vegetation features. In our analysis, this will overestimate the number of occurrences that are actually outside the fundamental niche, and thus underestimate the probability of a random occurrence having favorable climate. Therefore, the performed binomial test errs on the conservative side (more violations to Hutchinson’s predictions that actually taking place).

The estimates of the realized niches were obtained by fitting smooth kernels to the observed temperatures. Smooth kernels are simple niche models [42], and we decided to fit them only for species with at least 5 points (92 species). For each species the intersection of its smooth kernel with **E**(*t*, **G**) inside the corresponding **N**_F_ was calculated integrating numerically the minimum of the species kernel and the climatic kernel, and multiplying by the observed range of temperatures, per species [43]. This is an estimate of how much the occurrences distribution actually overlap with the available niche space. All processing was performed in R and *Mathematica*^™^.

## Results

**Fig 1:**
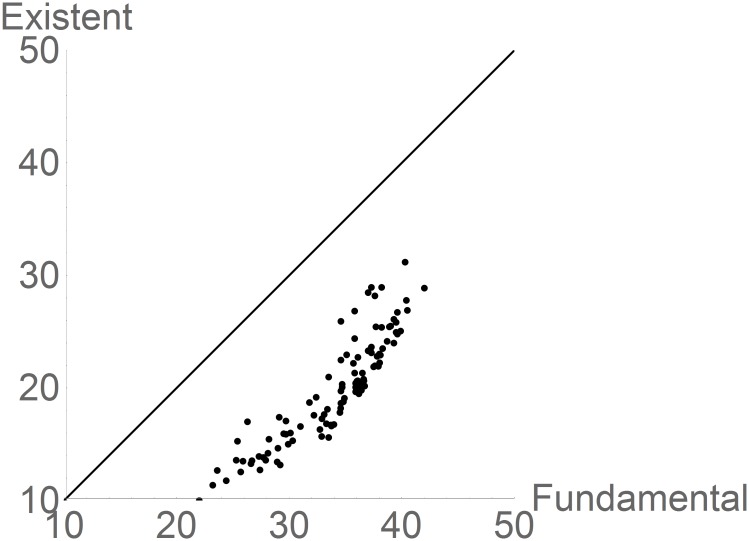
Scattergram (decimal logarithm) of the measure of the existing (N*(*t*, G)) versus the fundamental (N_F_) niches for 105 species. The units for **N**_F_ and N*(*t*, **G**) are in °C. The straight line is the line of Existing = Fundamental. The units of the existing niche can be transformed to units of area potentially suitable for each species. The distance between a point and the line is a measure of how much potential for evolving wider tolerance ranges a species has (see text).

Eq (1) states that **N**_*F*_ ⊇ N*(*t*, **G**). This is true by definition. However, a measure of how much larger **N**_F_ is than N*(*t*, **G**), for a specific time and region, is a measure of how much “unused” fundamental niche exists for a species, at a given time, and therefore how much geographic area would be left to be occupied, given the correspondence previously mentioned between metrics in niche and geographic space. This is shown in Fig 1. Roughly speaking, species occupy available favorably climates in proportion to their capacity, since N*(*t*; **G**) is proportional to **N**_F_ (N* = -11.5 + 0.925 **N**_F_, p << 0.01, r^2^ = 0.799). However, there is a gap between the identity line and the existing niches. This gap is a measure of how much the existing and favorable temperatures in the world are not used by a species, and this can also be interpreted as a measure of the potential to evolve wider temperature tolerances, given the actual temperature distributions. Hutchinson’s duality allows looking at the results from a geographic perspective: the number of grid cells with temperatures inside the existing niches, N*(*t*; **G**) is a measure of the size of the potential distribution and constitutes an upper bound to the size (number of pixels of 10^4^ km^2^ in geographic space) of the actual distribution of a species [12]. The range of the number of geographic cells with climates inside the critical/lethal ranges of the species in the database was [395, 2215], which implies that with the current climate, actual ranges larger than ~2.1 x 10^7^ km^2^ should not exist.

**Fig 2:**
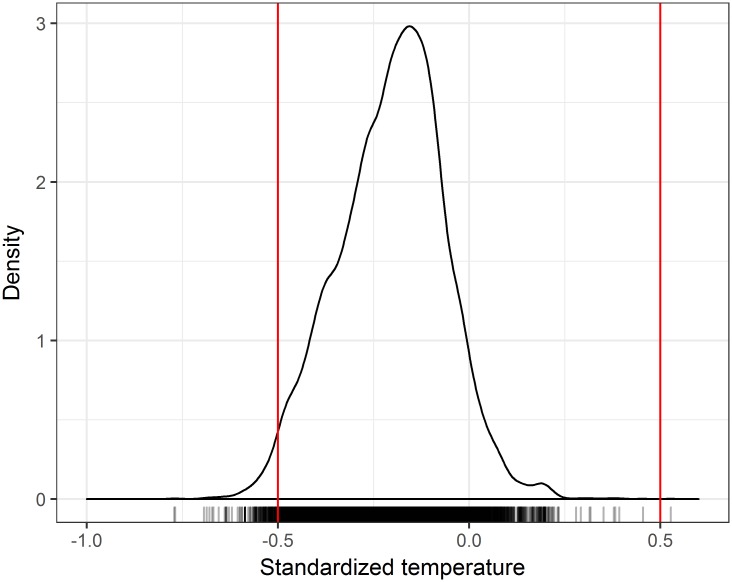
Proportion of GBIF points inside the N_F_ for a common scale of fundamental niches (bars) and smooth histogram of the distribution of observed standardized temperatures. The red bars show the limits of the 105 **N**_F_ in the common scale.

Eq (1) also states that climates in **N**_R_(*t*, **G**) should be inside of **N**_F_ (the original Hutchinson’s prediction). In total, 85.86% of the ~14000 observation records of all species had climates inside their corresponding **N**_F_ boxes, a result highly unlikely assuming random data on a uniform distribution (Fig 2). However, since every fundamental niche is different, and temperature is not uniformly distributed, for each species we estimated the probability that random sampling from **E**(*t*, **G**) would give the observed or a greater number of points inside the corresponding niche, with a 0.01 significance sequential Bonferroni correction for multiple comparisons (see Methods). When using species with at least five occurrences, 73 out of 92 species have more observations inside the limits of their **N**_F_ than expected by chance. Although 29 species have at least one observation outside its **N**_F_, at this level (0.05/151) no species has more observations outside its limits than expected by chance (Table A in S1 File).

**Fig 3:**
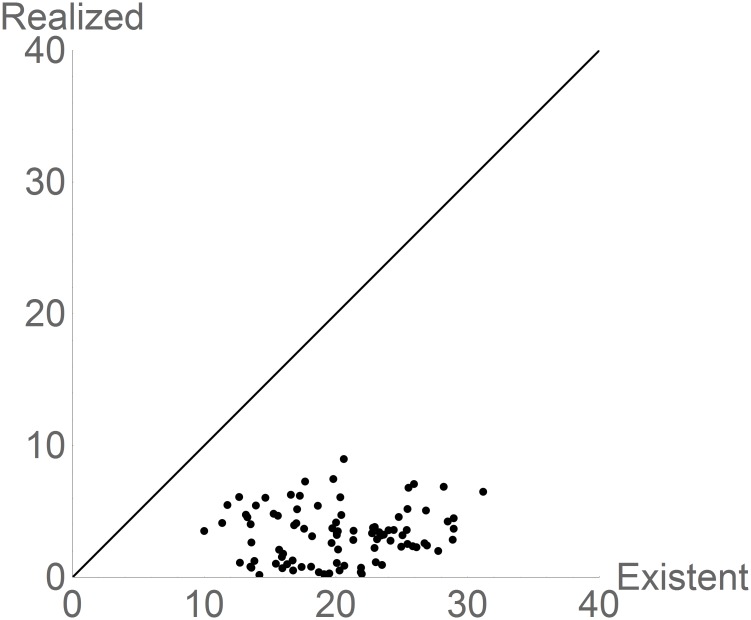
Scattergram (decimal logarithm) of the measure of N_R_(*t*, G) vs. N*(*t*, G) for 92 species with more than five occurrences. The units for **N**_R_(*t*, **G**) and N*(*t*, **G**) are in °C. The straight line is the line of Realized = Existing. The distance between a point and the line is a measure of how much favorable space is not being used by a species, maybe due to interactions, dispersal limitations or other such factors (see text).

The inequalities in Eq (2) predict that for every species, and at a given time *t* and region **G**, the measure of the **N**_R_(*t*, **G**) should be smaller than the measure of the corresponding N*(*t*, **G**). These two variables are displayed in Fig 3 showing that for most species the amount of realized niche space actually used, as estimated from the occurrences data, is almost constant, at about 2–10 degrees of temperature, but there are many species for which there is a very substantial amount of existing niche space that is not used. This is a measure of invasibility potential.

**Fig 4:**
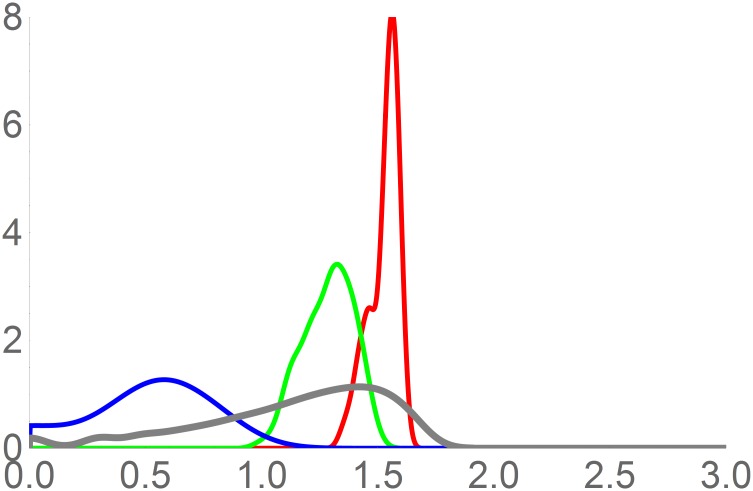
Smooth histograms of the decimal logarithm of values of a null model of fundamental niche (grey), the 105 values of fundamental (red), 105 existing (green) and 92 realized (blue).

In Fig 4 we present smooth histograms of all the niches described above, and of a null model, showing that the data of ranges of temperature of fundamental niches for reptiles and amphibians gives a much narrower and significantly different distribution (Kolmogorv-Smirnov, p < 10^−16^) than a random distribution of volumes.

## Discussion

Strictly speaking, only physiologically-obtained ranges of conditions suitable for species, should be used as measures of fundamental niches. Today, for more than one dimension, such measures are scarce, and our test then was performed on a limited number of species using a single niche variable. Although we used a single variable dataset, all the operations we described can be performed in two or more dimensions, assuming that information on multiple physiological limits is available. In S1 File, Section 2, we present an illustration of how to do this for two dimensional niches.

For the one-dimensional dataset we used, the results are very clear: both Hutchinson’s equations (sets and their measures) are valid for niches of reptiles and amphibians, based on temperature as niche variable, and GBIF occurrences. In order to get this result, we needed definitions of fundamental, existing and realized niches that are precise (mathematical) and operational, i.e., capable of being calculated with actual data. A disadvantage of this is that such definitions narrow the meanings of the terms used, but in return relationships and predictions become clearer. It is also important to realize that the original ideas of Hutchinson can be interpreted both as set inequalities, predicting “containment” (Eq 1), and magnitude inequalities, predicting size relationships (Eq 2) and these are independent in regards to the realized niche: the realized niche may not be a part of the fundamental and still its magnitude may be smaller than that of the realized. This is not the case in any of the species we analyzed, but it is possible in principle.

Figs 1 and 3 display the sizes of the inequalities Eq (2). By construction **N**_*F*_ ⊇ N*(*t*, **G**) is true (see Methods), although N*(*t*, **G**) ⊇ **N**_*R*_(*t*, **G**) can be empirically false. However the size of the difference in the measures of **N**_F_, N*(*t*, **G**) and **N**_R_(*t*, **G**) is very interesting. In the current climate, for all species there is a gap of approximately 11 degrees between their range of tolerances (**N**_F_) and the actual available range of temperatures, suggesting the possibility of the existence of selective pressures that could lead to evolution of the fundamental niches. On the other hand, most realized niches are well below their existing niche potential which suggests that for the species in this analysis there are large areas of the world, currently not reported as occupied in the GBIF database, but with the favorable temperatures. This is an index of invasibility potential.

It is possible for **N**_R_(*t*, **G**) to have parts outside its **N**_F_, in violation of Eq (1), although none of our species violate Eq (1) significantly. This has interesting implications for correlative niche modeling, since it suggests that for some species a correlative ENM will be a very poor estimate of the size of the existing niche, and confirming that niche estimates based on species occurrence should not, in general, be regarded as estimates of fundamental niches. The only situation in which this is the case is when the species has had access to all possible environmental conditions, unrestricted by barriers, dispersal disequilibrium, or negative interactions [44], a highly hypothetical situation. An obvious morale of this is that the output of correlative niche models, like Maxent, Bioclim or others should not be used to perform evolutionary analysis unless there is proof that confounding ecological, dispersal or climatic factors can be disregarded, or the evolutionary hypothesis is stated in a way that takes into account the preceding caveats. For instance, Hof et al. [45] explored whether there was a phylogenetic signal in the realized niches of the amphibians of the world, which they found to be the case. Their hypothesis was carefully designed to take into account the complications introduced by use of the realized niche, unfortunately, this is not always the case.

The data we used to estimate realized niches come from GBIF observations, which (assuming random sampling) can be regarded as data representing true realized niches. Even correcting for spatial autocorrelation by thinning data, the climate of most occurrences is located inside their corresponding **N**_F_. However, most points are grouped towards the cool side of the graph (Fig 2), both those inside or out of the **N**_F_ box. It is known that distributions of temperature requirements [46] commonly have sharp cut-offs at high temperatures and longer tails at low temperatures. Also, a recent analysis of the temperature tolerance of more than 2,500 plants and of endothermic and ectothermic animals [32] reported significantly higher variability in tolerance to lower than to upper thermal limits. Therefore, an asymmetric physiological tolerance (their **N**_F_) could explain the distribution of occurrences in Fig 2. However, the proportion of environmental space available on different regions around the **N**_F_ of the species is not uniform. The simplest explanation for the skewed distribution of the environments of the occurrence points then would be simply that the realized niches of the species we use are biased to the left of their fundamental niches because environmental space is lacking on the hotter parts (S1 File, Fig A1). This is an explanation based on the combination of the shape of **E**(*t*, **G**) and the location of the species’ **N**_F_, as Eq (1) would suggest it should be done, thus highlighting the importance of the existing niche, a seldom remembered concept. In fact, the hypothesis that larger range sizes correspond to larger “niche breadth,” [47], without making the distinctions among niche types that become obvious by using symbols and operational definitions, misses the critical component of the availability of niche variables, a point made long ago [9].

We did not document species with a significant number of occurrences outside their **N**_F_, however, this can indeed happen. Leaving aside the possibility that the databases we use may contain poor estimates of ranges, taxonomy mistakes, or biased or non-obvious georeferencing errors, there are four ways that we see for Hutchinson’s inequality Eq (1) to fail. (i) Some facilitating mechanisms, natural or anthropogenic [48] are operating. This could result in points occurring outside purely physiological limits due to interactions, an interesting theoretical possibility that has not been explored empirically, but it is unlikely for the species we use. Also, favorable microhabitats or microclimatic spots may be found inside cells with unfavorable climate, or by behavioural responses. Maybe some of the points outside **N**_F_ left limit in Fig 2 are examples of this possibility, something we cannot test. (ii) Niche evolution *sensu stricto* occurred and populations shifted their physiological limits relative to the Sunday et al. [25] baselines. This possibility can be checked experimentally, documenting variability in tolerance ranges over geographic space [49, 50]. (iii) Because a single **N**_F_ range summarizes just the mortality part of the life history of a species, differences in requirements across the life history were ignored, which could lead to misleading results if, for example, different stages in the life history have different physiological requirements [51]. In other words, points outside a respective **N**_F_ may be true sink populations [52] and not errors of the prediction. Finally, (iv) the **N**_F_ may not be a convex set but instead may contain “holes” [42, 53], creating unsuitable regions inside the extremes of reported tolerances. This possibility could arise if physiological tolerances are not mono-modal, which is unlikely, or if there is marked niche differentiation in subpopulations [49]. These factors would render the use of intervals, boxes, ellipsoids or other convex sets unsuitable for representing **N**_F_. Testing these hypotheses is outside the scope of this communication, but stating them highlight the fundamental interest of Hutchinson’s inequalities.

Notice that evolution of the fundamental niche may occur in its size, shape and position. In a recent work, Gouveia et al. [31] showed that the larval critical thermal maximum, a physiological proxy for the **N**_F_, correlates better with the realized niche position than with measures of niche size. The fact that the authors were able to show a highly significant explanation of **N**_R_ position as predicted by their **N**_F_ proxy is suggestive that inequality Eq (1) is also fulfilled in their system.

Generally speaking, the problem of estimating fundamental niches from basic physiology or experimental data is still a neglected field, restricted mostly to temperature as niche variable [25, 29, 31, 54, 55]. Although a few experimental multivariate attempts to assess **N**_F_ exist, [56, 57], an interesting alternative is to measure fundamental niches using the physiological models of Carbon and Nitrogen allocation in plants [58, 59] that would allow calculation of strict fundamental niches. Higgins and Richardson [60] fitted such models to observed occurrences of *Acacia* and *Eucalyptus* in Australia, and show that independent GBIF observation records are very well predicted by extrapolation from the physiological models, which is consistent with the predictions of Eq (1). Unfortunately, by fitting the physiological model to observed occurrences Higgins and Richardson [60] did not provide a strict test of Eq (1), since their fit was biased by processes other than physiological that affect observed records. On the other hand, Higgins and Richardson [60] also applied their fitted models to a hypothetical world with equally common environmental zones. This means a uniform distribution of variable values in **E** space, which by Eq (1) would imply they removed the reducing effects of anisotropic environmental space yielding values of N*(*t*, **G**) much larger than for the real world, a result that indeed is shown in their Fig 2 but is not elaborated in their discussion.

Our tests of niche inequalities mostly support their validity but required a restricted definition of niche based on a specific type of variable (scenopoetic climate). This definition is operational and enables the use of large and existing datasets, but most importantly, multivariate climate niches allow definitions to be based on straightforward set operations with direct relationships to area, which is a major advantage. Less restrictive definitions can be used to try to understand relations between niches and areas of distribution [47], but many complications may thus remain hidden. For instance, inequalities Eq (1) reveals the critical importance of considering the existing niche space as an integral part of niche analysis [9, 61]. The fundamental niche is expressed in specific environments which are dynamic. Our analysis shows how apparent patterns in distributions of realized niches may have as a parsimonious explanation the bias in the location of fundamental niches in the anisotropic environmental space.

Although our test of Hutchinson’s inequality was essentially successful, it relies on reducing the scope of meaning of terms, using only certain types of variables for the multivariate space, resorting to some mostly ignored concepts (existing niche), and stating explicitly a number of assumptions about fundamental niches (convexity, inequalities). These are steps in the development of a rigorous theory of Grinnellian niches, one that we think is much needed not only to provide a conceptual scaffolding to the burgeoning field of niche modeling, but also because it may end clarifying and illuminating many question in biogeography and macroecology.

## Supporting information

S1 File Supplementary figures, text describing niche operations in two dimensions, and table with basic data for 105 species. (DOCX)
