# Supplementary material for: Are fundamental niches larger than the realized? Testing a 50-year-old prediction by Hutchinson
Source: PLoS One. 2017 Apr 12;12(4):e0175138. doi: 10.1371/journal.pone.0175138 (PMC5389801; doi:10.1371/journal.pone.0175138)
Supplement: S1 File — (DOCX) [file pone.0175138.s001.docx]

Supporting Information for: Are fundamental niches larger than the realized? Soberón & Arroyo-Peña

1. **Figures**


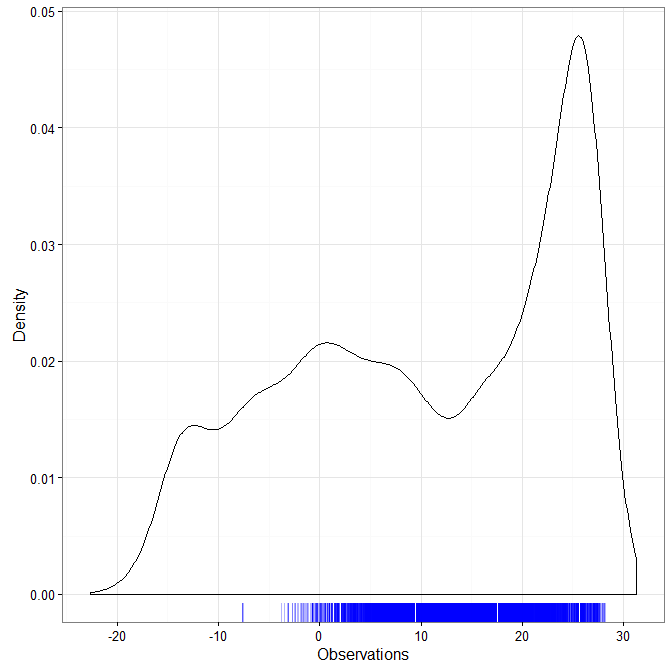


Figure A. Distribution of available temperature in the world (line) and observations from GBIF occurrences (blue rug). Notice that there is a wider availability of temperatures on the cold side of the graph.


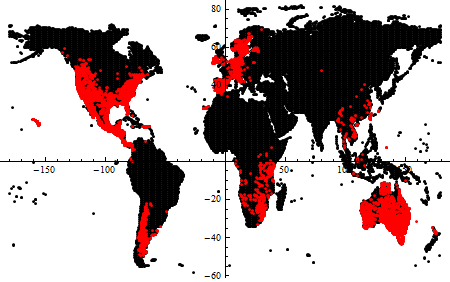


Figure B. The distribution of 14,075 GBIF points for the 105 species in the list of reptiles and amphibians taken from Sunday et al. ([2011](#_ENREF_3)).


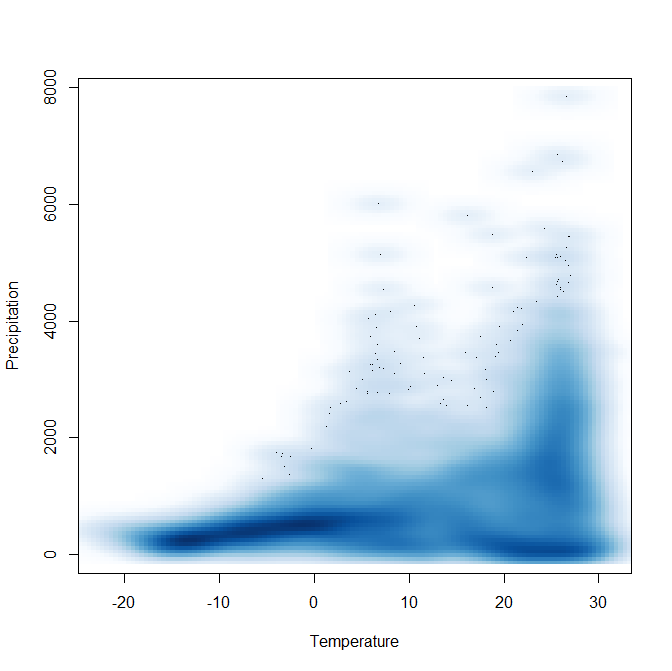


Fig. C. Smooth kernel of WorldClim (Bio1/10 and Bio12) fitted to 16,712 points in a regular grid of the emerged part of the planet (without Antarctica). Resolution 10’.

1. **Estimation of niches in two dimensions**

It is possible to fit smooth kernels in higher dimensions ([Blonder, Lamanna, et al. 2014](#_ENREF_1)). For instance, a smooth scatterplot, *kE*(*x,y*) fitted to environmental space in dimensions of mean temperature (centigrade) and annual precipitation (mm) is displayed in Fig A3. The function smoothScatterplot in R was used.

*Calculation of niche metrics*

The fundamental niche of a species is defined by all the points inside the rectangle of ranges (or inside any shape defining the fundamental niche) that allow the species to survive. For instance, if the shape is a rectangle defined over temperature and precipitation: $\mathbf{N}_{F_{j}}=\{(T,P)|\mathrm{T}_{\min}\leq T\leq T_{\max},P_{\min}\leq P\leq P_{\max}\}$, and a natural measure for it is simply the area of the rectangle: $|\mathbf{N}_{F_{j}}|=(T_{\max}-T_{\min})(P_{\max}-P_{\min})_{j}$. Notice that the fundamental niche is by definition a physiological property of a species, and to project it to geography, information about the structure of the variables in question, for a region, at a given time, is needed.

The existing niche is defined as the amount of actual niche space contained within the fundamental niche of a given species. Denoting by **E**(*t*, **G**) a set of environmental combinations (*x*, *y*) that are representative of a given region **G**, at a time *t*, then $\mathbf{N}_{F_{j}}^{*}=\{(x,y)\in\mathbf{E}(t,G)\cap\mathbf{N}_{F_{j}}\}$, and a possible measure is: $|\mathbf{N}^{*}_{F_{j}}|=|\mathbf{N}_{F_{j}}|\int_{\mathbf{N}_{F_{j}}} k\mathbf{E}=|\mathbf{N}_{F_{j}}|\int_{T_{\min}}^{T_{\max}} \int_{P_{\min}}^{P_{\max}} k\mathbf{E}(x,y)dxdy$ , with the limits taken for the tolerance ranges of each species. If the fundamental niche is defined by a non-rectangular shape, the integral is taken over the region defining$\mathbf{N}_{F_{j}}$. In any case the existing niche is the amount of actual niche space inside a given fundamental niche.

Finally, realized niches are defined as the smooth kernels around the occurrence points $\mathbf{R}_{j}(x,y)=kO_{j}(x,y)$ , and we use as a measure of the overlap between the two ([Stine and Heyse 2001](#_ENREF_2)) the integral of the minimum of *kE*(*x,y*) and *kO_j_*(*x,y*), taken over the entire range of existing climates. This is equivalent to say that a realized niches is the amount of existing climate inside the region actually occupied by a species: $|\mathbf{R}_{j}|=|\mathbf{N}_{F_{j}}|\int_{T_{\min}}^{T_{\max}} \int_{P_{\min}}^{P_{\max}} \min\left[kE(x,y),kO_{j}(x,y)\right]dxdy$.

**Table A.** Main information for the species used. Tmin and Tmax are the critical and/or lethal temperatures from Sunday et al. ([2011](#_ENREF_3)). GBIF is the number of localities from GBIF, thinned to 10km. In N_F_ is the number of localities with mean temperature inside the critical range [Tmin,Tmax], and Out N_F_ the number of points outside the range. Size N* are the values (in centigrade) of $(T_{\max}-T_{\min})\int_{T_{\min}}^{T_{\max}} kE(x)dx$. pIn is the probability of having a random point of the existing climate (line in Figure S1) inside a given fundamental niche. pInRnd is the binomial probability of the number of points In N_F_, given GBIF occurrences, with probability pIn. pOutRnd is the binomial probability of OutN_F_, given GBIF occurrences, with probability 1-pIn of being outside a fundamental niche. The grey highlighted species are those with five or more occurrences and a non-significant number of observations inside the fundamental niche.

| Class | Name | Tmin | Tmax | GBIF | In N_F_ | Out N_F_ | Size N_F_ | SizeN* | pIn | pInRnd | pOutRnd |
| --- | --- | --- | --- | --- | --- | --- | --- | --- | --- | --- | --- |
| Amph | Craugastor fleischmanni | 6 | 31.4 | 23 | 23 | 0 | 25.4 | 15.28 | 0.601 | 8.35E-06 | 1.00E+00 |
| Amph | Dendrobates auratus | 9.5 | 34.8 | 77 | 77 | 0 | 25.3 | 13.58 | 0.537 | 1.58E-21 | 1.00E+00 |
| Amph | Desmognathus fuscus | -2.3 | 35 | 247 | 247 | 0 | 37.3 | 28.93 | 0.776 | 5.67E-28 | 1.00E+00 |
| Amph | Eleutherodactylus coqui | 7.5 | 37.2 | 62 | 62 | 0 | 29.7 | 17.06 | 0.574 | 1.16E-15 | 1.00E+00 |
| Amph | Eurycea bislineata | -2 | 35 | 310 | 310 | 0 | 37 | 28.48 | 0.770 | 5.94E-36 | 1.00E+00 |
| Amph | Hyla walkeri | -2.2 | 38.1 | 28 | 28 | 0 | 40.3 | 31.18 | 0.774 | 7.60E-04 | 1.00E+00 |
| Amph | Pachymedusa dacnicolor | 2.5 | 28.8 | 122 | 122 | 0 | 26.3 | 17.01 | 0.647 | 7.91E-24 | 1.00E+00 |
| Amph | Pseudacris cadaverina | -1.4 | 36.8 | 112 | 112 | 0 | 38.2 | 28.94 | 0.758 | 3.18E-14 | 1.00E+00 |
| Amph | Pseudacris regilla | -1 | 36.6 | 226 | 226 | 0 | 37.6 | 28.18 | 0.749 | 4.90E-29 | 1.00E+00 |
| Amph | Rana cascadae | -1 | 33.6 | 106 | 106 | 0 | 34.6 | 25.93 | 0.749 | 5.19E-14 | 1.00E+00 |
| Amph | Rana pretiosa | -1 | 34.8 | 148 | 143 | 5 | 35.8 | 26.83 | 0.749 | 7.40E-13 | 1.00E+00 |
| Rept | Amphibolurus muricatus | 3 | 42.3 | 270 | 270 | 0 | 39.3 | 26.11 | 0.664 | 1.11E-48 | 1.00E+00 |
| Rept | Anolis carolinensis | 4.7 | 41.7 | 325 | 325 | 0 | 37 | 23.30 | 0.630 | 5.23E-66 | 1.00E+00 |
| Rept | Anolis cooki | 9.3 | 38.8 | 6 | 6 | 0 | 29.5 | 15.94 | 0.540 | 2.49E-02 | 1.00E+00 |
| Rept | Anolis cristatellus | 8.9 | 37.1 | 62 | 62 | 0 | 28.2 | 15.45 | 0.548 | 6.17E-17 | 1.00E+00 |
| Rept | Anolis cupreus | 11.8 | 38.4 | 50 | 50 | 0 | 26.6 | 13.27 | 0.499 | 7.97E-16 | 1.00E+00 |
| Rept | Anolis gundlachi | 6.3 | 35.4 | 24 | 24 | 0 | 29.1 | 17.40 | 0.598 | 4.34E-06 | 1.00E+00 |
| Rept | Anolis humilis | 12.4 | 35.6 | 53 | 53 | 0 | 23.2 | 11.36 | 0.490 | 3.71E-17 | 1.00E+00 |
| Rept | Anolis intermedius | 11.1 | 38.4 | 14 | 14 | 0 | 27.3 | 13.92 | 0.510 | 8.03E-05 | 1.00E+00 |
| Rept | Anolis lemurinus | 12.9 | 37.3 | 155 | 155 | 0 | 24.4 | 11.77 | 0.482 | 8.01E-50 | 1.00E+00 |
| Rept | Anolis lionotus | 14.7 | 36.7 | 20 | 20 | 0 | 22 | 9.99 | 0.454 | 1.39E-07 | 1.00E+00 |
| Rept | Anolis tropidolepis | 9.5 | 33.1 | 23 | 23 | 0 | 23.6 | 12.66 | 0.537 | 6.06E-07 | 1.00E+00 |
| Rept | Austrelaps superbus | 3 | 41.2 | 299 | 299 | 0 | 38.2 | 25.38 | 0.664 | 7.85E-54 | 1.00E+00 |
| Rept | Bassiana duperreyi | 3 | 43.5 | 223 | 223 | 0 | 40.5 | 26.91 | 0.664 | 2.47E-40 | 1.00E+00 |
| Rept | Chamaeleo dilepis | 7.6 | 43.6 | 358 | 357 | 1 | 36 | 20.60 | 0.572 | 4.62E-85 | 1.00E+00 |
| Rept | Ctenotus regius | 8.7 | 45.1 | 133 | 133 | 0 | 36.4 | 20.07 | 0.551 | 4.13E-35 | 1.00E+00 |
| Rept | Ctenotus taeniolatus | 11.4 | 44.7 | 190 | 187 | 3 | 33.3 | 16.82 | 0.505 | 4.78E-51 | 1.00E+00 |
| Rept | Ctenotus uber | 9.1 | 45.5 | 195 | 195 | 0 | 36.4 | 19.80 | 0.544 | 2.81E-52 | 1.00E+00 |
|  |  |  |  |  |  |  |  |  |  |  |  |
| Class | Name | Tmin | Tmax | GBIF | In N_F_ | Out N_F_ | Size N_F_ | Size N* | Prob IN | Prob INRnd | Prob OutRnd |
| Rept | Dipsosaurus dorsalis | 14 | 47.5 | 250 | 246 | 4 | 33.5 | 15.59 | 0.465 | 2.52E-75 | 1.00E+00 |
| Rept | Egernia cunninghami | 4.7 | 41.9 | 307 | 307 | 0 | 37.2 | 23.42 | 0.630 | 2.16E-62 | 1.00E+00 |
| Rept | Egernia saxatilis | 4.5 | 41.8 | 223 | 223 | 0 | 37.3 | 23.64 | 0.634 | 6.65E-45 | 1.00E+00 |
| Rept | Egernia striolata | 6.1 | 44.2 | 234 | 234 | 0 | 38.1 | 22.93 | 0.602 | 2.46E-52 | 1.00E+00 |
| Rept | Eremias brenchleyi | 5.1 | 40.8 | 2 | 2 | 0 | 35.7 | 22.19 | 0.622 | 3.86E-01 | 1.00E+00 |
| Rept | Eremiascincus fasciolatus | 9 | 41.2 | 172 | 172 | 0 | 32.2 | 17.58 | 0.546 | 6.02E-46 | 1.00E+00 |
| Rept | Eremiascincus richardsonii | 7.3 | 42 | 195 | 195 | 0 | 34.7 | 20.06 | 0.578 | 3.97E-47 | 1.00E+00 |
| Rept | Eulamprus kosciuskoi | 2.5 | 40.2 | 61 | 61 | 0 | 37.7 | 25.44 | 0.675 | 3.79E-11 | 1.00E+00 |
| Rept | Gehyra variegata | 6 | 43.8 | 290 | 290 | 0 | 37.8 | 22.82 | 0.604 | 2.84E-64 | 1.00E+00 |
| Rept | Hemiergis decresiensis | 9.6 | 39.3 | 129 | 128 | 1 | 29.7 | 15.89 | 0.535 | 1.05E-33 | 1.00E+00 |
| Rept | Hemiergis peronii | 6.8 | 38.6 | 167 | 167 | 0 | 31.8 | 18.70 | 0.588 | 3.03E-39 | 1.00E+00 |
| Rept | Heteronotia binoei | 9.6 | 40.6 | 304 | 304 | 0 | 31 | 16.59 | 0.535 | 2.76E-83 | 1.00E+00 |
| Rept | Lacerta agilis | 5.9 | 43.9 | 274 | 259 | 15 | 38 | 23.02 | 0.606 | 7.45E-39 | 1.00E+00 |
| Rept | Lampropholis delicata | 4.7 | 40.8 | 199 | 199 | 0 | 36.1 | 22.73 | 0.630 | 1.07E-40 | 1.00E+00 |
| Rept | Lepidophyma flavimaculatum | 11.3 | 38 | 117 | 117 | 0 | 26.7 | 13.53 | 0.507 | 2.86E-35 | 1.00E+00 |
| Rept | Liolaemus bibronii | 8.49 | 44.5 | 137 | 77 | 60 | 36.04 | 20.01 | 0.555 | 4.72E-01 | 9.98E-01 |
| Rept | Liolaemus boulengeri | 9.4 | 44 | 46 | 27 | 19 | 34.59 | 18.63 | 0.539 | 3.06E-01 | 9.68E-01 |
| Rept | Liolaemus canqueli | 6.99 | 44.6 | 5 | 5 | 0 | 37.61 | 21.97 | 0.584 | 6.81E-02 | 1.00E+00 |
| Rept | Liolaemus chacoensis | 14.9 | 44.1 | 17 | 14 | 3 | 29.2 | 13.17 | 0.451 | 1.91E-03 | 9.96E-01 |
| Rept | Liolaemus cuyanus | 12 | 45.7 | 6 | 6 | 0 | 33.7 | 16.71 | 0.496 | 1.49E-02 | 1.00E+00 |
| Rept | Liolaemus darwinii | 10.1 | 44.7 | 126 | 119 | 7 | 34.57 | 18.19 | 0.526 | 3.19E-25 | 1.00E+00 |
| Rept | Liolaemus fitzingerii | 8.24 | 44.5 | 16 | 15 | 1 | 36.21 | 20.28 | 0.560 | 1.27E-03 | 1.00E+00 |
| Rept | Liolaemus irregularis | 6.96 | 45 | 3 | 2 | 1 | 38.02 | 22.23 | 0.585 | 6.26E-01 | 9.28E-01 |
| Rept | Liolaemus kingii | 7.01 | 44.5 | 14 | 14 | 0 | 37.49 | 21.89 | 0.584 | 5.35E-04 | 1.00E+00 |
| Rept | Liolaemus koslowskyi | 13.2 | 46.1 | 24 | 22 | 2 | 32.88 | 15.70 | 0.477 | 7.03E-06 | 1.00E+00 |
| Rept | Liolaemus kriegi | 7.6 | 43.7 | 37 | 20 | 17 | 36.07 | 20.64 | 0.572 | 7.13E-01 | 9.39E-01 |
| Rept | Liolaemus laurenti | 12.2 | 46.2 | 5 | 5 | 0 | 33.98 | 16.75 | 0.493 | 2.91E-02 | 1.00E+00 |
| Rept | Liolaemus melanops | 5.65 | 44.9 | 24 | 23 | 1 | 39.29 | 24.00 | 0.611 | 1.18E-04 | 1.00E+00 |
| Rept | Liolaemus multicolor | 8.06 | 44.7 | 3 | 1 | 2 | 36.64 | 20.65 | 0.563 | 9.17E-01 | 5.95E-01 |
| Rept | Liolaemus multimaculatus | 8.93 | 43.8 | 11 | 11 | 0 | 34.9 | 19.10 | 0.547 | 1.32E-03 | 1.00E+00 |
| Rept | Liolaemus olongasta | 11.9 | 44.6 | 16 | 15 | 1 | 32.73 | 16.30 | 0.498 | 2.46E-04 | 1.00E+00 |
| Rept | Liolaemus petrophilus | 7.31 | 45.2 | 18 | 17 | 1 | 37.92 | 21.92 | 0.578 | 7.32E-04 | 1.00E+00 |
| Rept | Liolaemus poecilochromus | 9.35 | 44.2 | 1 | 1 | 0 | 34.81 | 18.78 | 0.540 | 5.40E-01 | 1.00E+00 |
| Rept | Liolaemus pseudoanomalus | 12.2 | 45.9 | 1 | 1 | 0 | 33.74 | 16.63 | 0.493 | 4.93E-01 | 1.00E+00 |
| Rept | Liolaemus quilmes | 8.46 | 44.7 | 20 | 17 | 3 | 36.23 | 20.14 | 0.556 | 5.71E-03 | 1.00E+00 |
| Rept | Liolaemus riojanus | 10.7 | 45.2 | 1 | 1 | 0 | 34.5 | 17.81 | 0.516 | 5.16E-01 | 1.00E+00 |
| Rept | Liolaemus robertmertensi | 14.1 | 43 | 2 | 2 | 0 | 28.93 | 13.43 | 0.464 | 2.16E-01 | 1.00E+00 |
| Rept | Liolaemus rothi | 5.5 | 43.8 | 41 | 41 | 0 | 38.3 | 23.51 | 0.614 | 2.03E-09 | 1.00E+00 |
| Rept | Liolaemus scapularis | 9.39 | 45.6 | 7 | 7 | 0 | 36.18 | 19.49 | 0.539 | 1.32E-02 | 1.00E+00 |
| Rept | Liolaemus xanthoviridis | 7.86 | 44.5 | 1 | 1 | 0 | 36.62 | 20.77 | 0.567 | 5.67E-01 | 1.00E+00 |
| Class | Name | Tmin | Tmax | GBIF | In N_F_ | Out N_F_ | Size N_F_ | Size N* | Prob IN | Prob INRnd | Prob OutRnd |
| Rept | Moloch horridus | 3.5 | 42.5 | 354 | 354 | 0 | 39 | 25.51 | 0.654 | 5.25E-66 | 1.00E+00 |
| Rept | Notechis scutatus | 2.2 | 38 | 392 | 392 | 0 | 35.8 | 24.38 | 0.681 | 4.18E-66 | 1.00E+00 |
| Rept | Phymaturus patagonicus | 9.8 | 39.9 | 9 | 2 | 7 | 30.1 | 16.00 | 0.532 | 9.88E-01 | 1.25E-01 |
| Rept | Platysaurus intermedius | 8.9 | 44.8 | 135 | 135 | 0 | 35.9 | 19.66 | 0.548 | 5.06E-36 | 1.00E+00 |
| Rept | Plestiodon gilberti | 7.7 | 42.3 | 219 | 212 | 7 | 34.6 | 19.74 | 0.570 | 2.50E-42 | 1.00E+00 |
| Rept | Podarcis muralis | 8.3 | 44.2 | 162 | 137 | 25 | 35.9 | 20.06 | 0.559 | 6.45E-15 | 1.00E+00 |
| Rept | Protobothrops mucrosquamatus | 3.5 | 38.6 | 28 | 28 | 0 | 35.1 | 22.96 | 0.654 | 6.86E-06 | 1.00E+00 |
| Rept | Psammodromus algirus | 7 | 43.5 | 852 | 848 | 4 | 36.5 | 21.32 | 0.584 | 5.68E-190 | 9.99E-01 |
| Rept | Psammodromus hispanicus | 8.8 | 45.5 | 839 | 835 | 4 | 36.7 | 20.17 | 0.550 | 6.86E-209 | 1.00E+00 |
| Rept | Pseudemoia entrecasteauxii | 2.5 | 42.1 | 69 | 69 | 0 | 39.6 | 26.72 | 0.675 | 1.63E-12 | 1.00E+00 |
| Rept | Pseudemoia spenceri | 1.9 | 42.3 | 137 | 137 | 0 | 40.4 | 27.77 | 0.687 | 5.06E-23 | 1.00E+00 |
| Rept | Pseudonaja textilis | 3.5 | 42.4 | 518 | 518 | 0 | 38.9 | 25.44 | 0.654 | 3.00E-96 | 1.00E+00 |
| Rept | Sceloporus graciosus | 4.8 | 44.7 | 175 | 163 | 12 | 39.9 | 25.04 | 0.628 | 1.02E-20 | 1.00E+00 |
| Rept | Sceloporus malachiticus | 9.7 | 42.8 | 97 | 97 | 0 | 33.1 | 17.65 | 0.533 | 3.30E-27 | 1.00E+00 |
| Rept | Sceloporus merriami | 14.1 | 41.5 | 66 | 66 | 0 | 27.4 | 12.71 | 0.464 | 9.52E-23 | 1.00E+00 |
| Rept | Sceloporus occidentalis | 4.6 | 44.1 | 188 | 188 | 0 | 39.5 | 24.95 | 0.632 | 3.14E-38 | 1.00E+00 |
| Rept | Sceloporus undulatus | 11.4 | 40.4 | 302 | 249 | 53 | 29 | 14.65 | 0.505 | 5.80E-31 | 1.00E+00 |
| Rept | Sceloporus variabilis | 10.2 | 43.1 | 127 | 127 | 0 | 32.9 | 17.26 | 0.525 | 2.68E-36 | 1.00E+00 |
| Rept | Sphaerodactylus gaigeae | 12.5 | 38.2 | 1 | 1 | 0 | 25.7 | 12.55 | 0.488 | 4.88E-01 | 1.00E+00 |
| Rept | Sphaerodactylus klauberi | 10.4 | 36.3 | 15 | 15 | 0 | 25.9 | 13.50 | 0.521 | 5.71E-05 | 1.00E+00 |
| Rept | Sphaerodactylus macrolepis | 11.8 | 39.5 | 14 | 14 | 0 | 27.7 | 13.82 | 0.499 | 5.92E-05 | 1.00E+00 |
| Rept | Sphaerodactylus nicholsi | 12.6 | 40.5 | 17 | 17 | 0 | 27.9 | 13.58 | 0.487 | 4.83E-06 | 1.00E+00 |
| Rept | Sphaerodactylus roosevelti | 11.4 | 39.5 | 5 | 5 | 0 | 28.1 | 14.19 | 0.505 | 3.29E-02 | 1.00E+00 |
| Rept | Sphaerodactylus townsendi | 11.4 | 41.7 | 4 | 4 | 0 | 30.3 | 15.31 | 0.505 | 6.51E-02 | 1.00E+00 |
| Rept | Sphenomorphus incognitus | 11.6 | 41.5 | 4 | 4 | 0 | 29.9 | 15.01 | 0.502 | 6.35E-02 | 1.00E+00 |
| Rept | Sphenomorphus taiwanensis | 4.9 | 38.4 | 1 | 1 | 0 | 33.5 | 20.96 | 0.626 | 6.26E-01 | 1.00E+00 |
| Rept | Takydromus septentrionalis | 4.9 | 44.5 | 11 | 11 | 0 | 39.6 | 24.78 | 0.626 | 5.75E-03 | 1.00E+00 |
| Rept | Takydromus sexlineatus | 6.4 | 42.2 | 49 | 49 | 0 | 35.8 | 21.33 | 0.596 | 9.59E-12 | 1.00E+00 |
| Rept | Tarentola boettgeri | 9.2 | 42.6 | 19 | 19 | 0 | 33.4 | 18.11 | 0.542 | 8.90E-06 | 1.00E+00 |
| Rept | Tiliqua nigrolutea | 5.2 | 42.5 | 266 | 266 | 0 | 37.3 | 23.11 | 0.620 | 5.23E-56 | 1.00E+00 |
| Rept | Tiliqua rugosa | 3.5 | 43 | 373 | 373 | 0 | 39.5 | 25.83 | 0.654 | 1.65E-69 | 1.00E+00 |
| Rept | Trimeresurus gracilis | 3.7 | 38.3 | 1 | 1 | 0 | 34.6 | 22.49 | 0.650 | 6.50E-01 | 1.00E+00 |
| Rept | Uta stansburiana | 7.8 | 43.7 | 268 | 267 | 1 | 35.9 | 20.41 | 0.568 | 3.72E-64 | 1.00E+00 |
| Rept | Varanus varius | 5 | 43.7 | 270 | 270 | 0 | 38.7 | 24.14 | 0.624 | 4.38E-56 | 1.00E+00 |
| Rept | Xantusia riversiana | 6.6 | 39 | 5 | 5 | 0 | 32.4 | 19.18 | 0.592 | 7.27E-02 | 1.00E+00 |
| Rept | Xantusia vigilis | 6.9 | 41.6 | 172 | 169 | 3 | 34.7 | 20.33 | 0.586 | 3.60E-35 | 1.00E+00 |
| Rept | Zootoca vivipara | 1.9 | 43.9 | 543 | 517 | 26 | 42 | 28.87 | 0.687 | 1.04E-53 | 1.00E+00 |

**Literature cited**

Blonder, B., et al. 2014. The n-dimensional hypervolume. - Global Ecology and Biogeography 23: 595-609.

Stine, R. A., et al. 2001. Non‐parametric estimates of overlap. - Statistics in medicine 20: 215-236.

Sunday, J. M., et al. 2011. Global analysis of thermal tolerance and latitude in ectotherms. - Proceedings of the Royal Society of London B: Biological Sciences 278: 1823-1830.
